# Rice transposable elements are characterized by various methylation environments in the genome

**DOI:** 10.1186/1471-2164-8-469

**Published:** 2007-12-20

**Authors:** Miwako Takata, Akihiro Kiyohara, Atsuko Takasu, Yuji Kishima, Hisako Ohtsubo, Yoshio Sano

**Affiliations:** 1 Laboratory of Plant Breeding, Graduate School of Agriculture, Hokkaido University, Sapporo 060-8589, Japan; 2 Institute of Molecular and Cellular Biosciences, University of Tokyo, Bunkyo-ku, Tokyo 113-0032, Japan

## Abstract

**Background:**

Recent studies using high-throughput methods have revealed that transposable elements (TEs) are a comprehensive target for DNA methylation. However, the relationship between TEs and their genomic environment regarding methylation still remains unclear. The rice genome contains representatives of all known TE families with different characteristics of chromosomal distribution, structure, transposition, size, and copy number. Here we studied the DNA methylation state around 12 TEs in nine genomic DNAs from cultivated rice strains and their closely related wild strains.

**Results:**

We employed a transposon display (TD) method to analyze the methylation environments in the genomes. The 12 TE families, consisting of four class I elements, seven class II elements, and one element of a different class, were differentially distributed in the rice chromosomes: some elements were concentrated in the centromeric or pericentromeric regions, but others were located in euchromatic regions. The TD analyses revealed that the TE families were embedded in flanking sequences with different methylation degrees. Each TE had flanking sequences with similar degrees of methylation among the nine rice strains. The class I elements tended to be present in highly methylated regions, while those of the class II elements showed widely varying degrees of methylation. In some TE families, the degrees of methylation were markedly lower than the average methylation state of the genome. In two families, dramatic changes of the methylation state occurred depending on the distance from the TE.

**Conclusion:**

Our results demonstrate that the TE families in the rice genomes can be characterized by the methylation states of their surroundings. The copy number and degree of conservation of the TE family are not likely to be correlated with the degree of methylation. We discuss possible relationships between the methylation state of TEs and their surroundings. This is the first report demonstrating that TEs in the genome are associated with a particular methylation environment that is a feature of a given TE.

## Background

Cytosine methylation is not only responsible for regulation of gene expression and immobilization of transposable elements (TEs) [1,2], but also gives cues about genomic environments [3]. In eukaryotic chromosomes, condensed regions such as the centromere and heterochromatin have intensively accumulated TEs, resulting in heavy DNA methylation in organisms that possess methylation machinery [4]. Recent genome-wide analyses using high-throughput methods revealed that plant genomes show differential methylation states between genes and repetitive sequences: gene-rich regions are present in the hypomethylated fraction, while repetitive sequences are often found in the hypermethylated fraction of the genome [5-7]. A comparative study of methylation status between *Arabidopsis *ecotypes revealed that TEs are commonly methylated, while genic methylation is highly polymorphic [8]. On the other hand, in mammalian genomes, genes and repeats did not show differential DNA methylation states [9]. Every repetitive sequence has a unique set of features regarding structure, transpositional manner, size, copy number, etc. Tran *et al*. [10] reported that the major epigenetic systems (DNA methylation and histone H3 lysine-9) in the *Arabidopsis *genome target TEs in a genome-wide manner, irrespective of element type and position. However, plant genomes contain an enormous number of repetitive sequences that are widely distributed over chromosomal segments that include hypomethylated euchromatic regions [11,12]. Little is known about the detailed methylation states of the regions around assorted repetitive sequences in plant genomes. We are interested in the methylation state surrounding the various types of plant repetitive sequences.

According to the decoded sequences [13], the rice genome comprises 389 mega base pairs (Mb) in total nucleotide length, and is one of the most compact genomes in monocotyledonous species. Despite its small genome size, TEs account for at least 35% of the total genome in rice. Representatives from all known TEs exist in the rice genome. The TEs in the rice genome consist mainly of two types of elements categorized into class I and class II, whose contents account for 20% and 13%, respectively, of the total length of the rice genome. Class I elements, including long terminal-repeat retrotransposons (LTR elements) and non-LTR retrotransposons (longand short-interspersed nucleotide elements: LINEs and SINEs), which transpose in the copy-and-paste manner. In particular, LTR elements are largely localized in gene-poor, heterochromatic regions such as the centromeric and pericentromeric regions [13]. Class II elements, which transpose in the cut-and-paste manner, outnumber class I elements by more than two fold, and often appear in euchromatic regions. The average size of class II elements is less than 300 bp, while that of class I elements is greater than 1000 bp, indicating that a large number of class II elements are scattered in the rice genome [13]. These differentially distributed TEs should reside in various differen genomic environments, particularly in terms of DNA methylation.

Previously, Takata *et al*. [14] detected DNA methylation in the flanking regions of two miniature inverted repeat transposable elements (MITEs) in AA-genome *Oryza *species using a transposon-display (TD) method. The proportion of methylated fragments in the total number of fragments was clearly different in the two MITEs. The methylation frequency estimated for one of the two MITEs was much lower than the average methylation frequency of the entire genomes of *Oryza *species. The results implied that MITEs show different preferences of the methylation state in their flanking sequences. This led us to pose the question of whether the sequences around TEs are hypermethylated or in specific methylation states.

In order to address the question, here we performed TD analyses targeting 12 rice TEs, which include class I, II, and other-class elements. The degrees of methylation of the TE flanking sequences were investigated by comparing the TD profiles between *Msp*I and *Hpa*II-digested samples. The 12 TEs tested here were found to show different features with regard to chromosomal distribution, copy number, and degree of conservation within a cognate family. We attempted to find relationships between the methylation state in the sequences adjacent to the TE and copy number or degree of conservation within the cognate family. The individual TE families reside in different genomic segments with varying degrees of methylation, but each family is present in an environment with a particular degree of methylation.

## Results and Discussion

### Selection of rice TEs

To examine the methylation states of the sequences flanking various repetitive elements, we selected 12 rice TEs that were classified into three groups: class I, class II, and a third group containing unknown type elements (Table 1). The representative sequences for the individual TEs examined here were those published in the papers listed in Table 1. Of the five elements in class I, *noaCRR1 *[15], *RIRE5 *(Taguchi and Otsubo unpublished result), and *RIRE7 *[16] belong to the LTR elements, and *p-SINE1 *[17] is a representative of rice *SINE*s. Two *Ty3/gypsy *type elements, *noaCRR1 *and *RIRE7*, were distributed around the centromeres. *RIRE5*, which is a member of the *Ty1/copia *type elements, was broadly distributed over the rice chromosomes, but not in the centromere. *p-SINE1 *is small sized (less than 500 bp) and was distributed randomly in euchromatic regions.

**Table 1:** Copy numbers and conservation degrees of 12 transposable elements based on the BLAST analyses

TE	Accession or reference (1)	Size (Pb)	Total count of Blast (A) (2)	Highest score (bit) (3)	Count over 80% of the highest score (B) (4)	80% of highest score	Conservation degree (B/A%) (5)
noaCRR1	AY827986	794	479	1467	29	1173	6.05
RIRE5	AB105803LTR	995	270	1674	14	1339	5.19
RIRE7	AB033234LTR	858	119	1541	30	1232	25.21
p-SINE1	Mochizuki et al. (1992) p-SINE1 consensus	123	207	209	66	167	31.88
Akan	AB077838	486	110	784	47	627	42.73
Kiddo	AF484680	269	42	459	37	367	88.1
Kiserul	AB077834	259	835	453	390	362	46.71
mPing	BK000588	430	50	795	48	636	96
Mashu	AB077839	263	563	457	441	360	78.33
Tabitoll	AB077831	146	563	226	339	180	60.21
Toya	AB077842	176	11	287	8	229	72.73
Basho	Inukai and Sano (2002) MS399	201	396	359	29	287	7.32

1: Representatives of the 12 TEs were selected from these accessions or references, which were used as the queries for the BLAST analyses.

2: These correspond to numbers of the sequences hit by the rice BLAST analyses at NCBI as described in Materials and Methods.

3: The scores are those of the most homologous sequences hit by the BLAST analyses.

4: The numbers of the sequences with more than 80% of the highest scores.

5: Conservation degrees of the TEs are estimated by percentages of the counts with more than 80% of the highest scores(B) in the total counts (A).

The Class II elements tested here consisted of *Akan *[18], *Kiddo *[19], *Kiseru *[18], *mPing *[20-22], *Mashu *[18], *TabitoII *[18], and *Toya *[18], all of which are miniature inverted repeat transposable elements (MITEs). Although *mPing *and *TabitoII *are affiliated with the *Tourist *family, the two structures are markedly different. *Toya *may constitute a large group of plant TEs, because elements that resemble *Toya *have been discovered in the tobacco [23] and wheat genomes [24]. For *Akan*, *Kiddo*, and *Mashu*, no counterparts have been identified so far. The class II elements in rice are dominated by the remarkable range of MITEs. The sequences for *Basho*, belonging to the unknown group [25], are present in certain portions of the rice genome, for instance Turcotte *et al*. [26] estimated the proportion of the unknown TE-like sequences in 910 kb of rice genomic DNA sequences to be 1.3%. BLAST searches did not find any specific features of the distribution patterns of the six MITEs and *Basho *in rice chromosomes, and they appeared to be dispersed over all euchromatic regions (data not shown).

### Copy number and conservation of TE sequences

Copy number is an important factor when one considers the cause of homology-dependent DNA methylation. However, it is difficult to estimate the precise numbers of repetitions even if the complete database is available. This is because most of the repetitive sequences have diversified during evolution. The older elements are more differentiated within the cognate sequences than the new elements. In order to survey the copy number and the degree of sequence conservation for the 12 TEs, we performed BLAST searches specifying the rice genome ([27] as of April, 2007), which takes account of gapped alignments between the query and subject sequences (Table 1). The outcomes of the BLAST searches using the representative sequences published for the 12 TEs as the queries revealed the total hit numbers, alignments of the hit sequences, and the bit scores. The total hit numbers of the 12 TEs were widely distributed from 11 to 835 (Table 1). We estimated the degrees of conservation in the individual TE families indicating the proportion of the conserved copy that was designated over 80% of the maximum bit score in the BLAST search using the most representative sequence, i.e., the 10% conservation degree does not mean 10% identity, but means that 10% of the total copy number in the TE family possesses more than 80% of the maximum bit score. The degree of conservation of the TEs analyzed here varied widely, ranging from 5% to 96%. Low conservation was seen in *noaCRR1*, *RIRE5*, and *Basho*, while the conserved copies of *mPing*, which is an active element in rice, accounted for 96% of the total copy number (Table 1). The information about the copy number and conservation degree was used for consideration of the relationships between DNA methylation and TEs.

### Transposon-displays for the cultivated and wild rice strains

The detection of methylation in TE flanking sequences was performed by the TD method based on amplified fragment length polymorphisms (AFLP), which is basically similar to that described by Takata *et al*. [14]. We designed the primers taking account of the conserved region within the 60-bp consensus sequences for the 12 TEs (see Additional file 1). All the 60-bp consensus sequences are located in the 5' end of the TEs. The detection of methylated fragments by TD requires a comparison between DNAs digested with methylation-sensitive and insensitive-restriction enzyme isoschizomers, e.g., *Hpa*II and *Msp*I, which recognize the same tetranucleotide sequence (5'-CCGG-3'). Hence, we surveyed the presence of cytosine methylation at 5'-CCGG-3' sites, which were located upstream of the TE copies. If a fragment from an *Msp*I-digested sample was located at the same position as that from the *Hpa*II-digested sample, the genomic segment was considered to be unmethylated. A fragment was considered to be methylated when the fragment derived from the *Msp*I-digested sample did not correspond to that from the *Hpa*II-digested sample. Nine rice genomes from Japonica, Indica, and *O. rufipogon *were tested in this study, all of which are closely related to each other, and whose hybrids produce fertile seeds. Evaluation of the nine strains was of benefit to verify the efficacy of the TD method for the detection of genome wide methylation. In the analyses, the same gel profile was reproduced in three independent trials of each primer pair.

**Figure 1:**
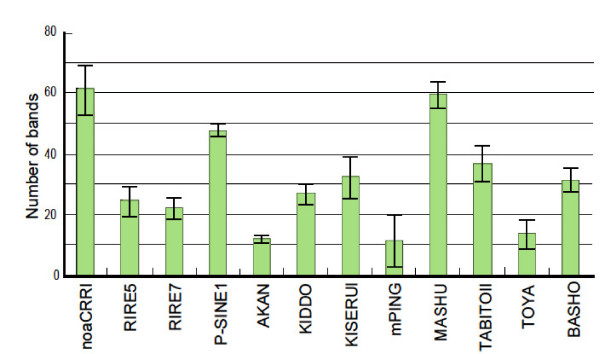
**Average numbers of bands obtained by TD analyses for the 12 rice TEs**. Each column shows the average number of bands that were estimated from the TD analyses of the nine cultivated and wild rice strains. The TD bands smaller than 500 bp in the *Msp*I-digested samples were counted in this study. Standard deviations were calculated from the data from the nine rice strains.

Because of the ambiguity of fragments larger than 500 bp detected by the TD method, only fragments below 500 bp were considered valid for evaluation in this study. The TD analyses showed different numbers of bands among the 12 TEs (Figure 1), reflecting the copy number of the TE family and its degree of conservation. In all the TEs examined here except *mPing*, the numbers of fragments below 500 bp detected in the *Msp*I-digested samples were not significantly different among the nine strains examined (Figure 1). Although the TD for *mPing *produced 27 - 38 bands below 500 bp in Japonica strains, as expected from the rice genome database, limited numbers (11-4) of bands for *mPing *were detectable in Indica and *O. rufipogon *strains. Indeed, it has been reported that Indica strains possess lower copy numbers of *mPing *than Japonica strains [20,21].

### Detection of DNA methylation

**Figure 2:**
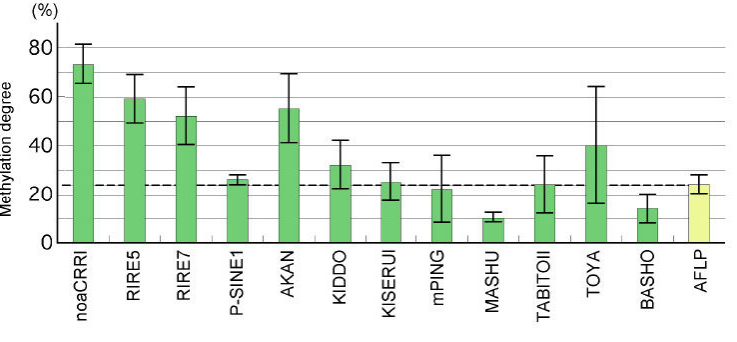
**Methylation degrees in the 5' flanking sequences of the 12 rice TEs**. The TD bands smaller than 500 bp in the *Msp*I-digested samples were compared with those in the corresponding *Hpa*II-digested samples. Methylation degrees are expressed as the proportion of the methylated bands in the total bands of the *Msp*I-digested samples. The average methylation degree (24.3%) for the whole genomes from the nine strains was estimated by AFLP analyses as described in the Methods, and is indicated by a dotted line. Standard deviations were calculated with the data from the nine rice strains.

In order to define the average degrees of cytosine methylation in the whole genomes, the AFLP method was employed using two sets of the digested samples (*Hpa*II/*EcoR*I and *Msp*I/*EcoR*I) for the nine genomes from the cultivated and wild rice strains, which were the same materials as used here. The proportion of methylated fragments in the total number of fragments detected by AFLP ranged between 23% and 28% among the nine genomes (Figure 2). The results implied that an average of 24% of the CCGG motifs in the examined genomes were methylated, as the distribution of *Eco*RI sites was not strongly influenced by methylation.

**Figure 3:**
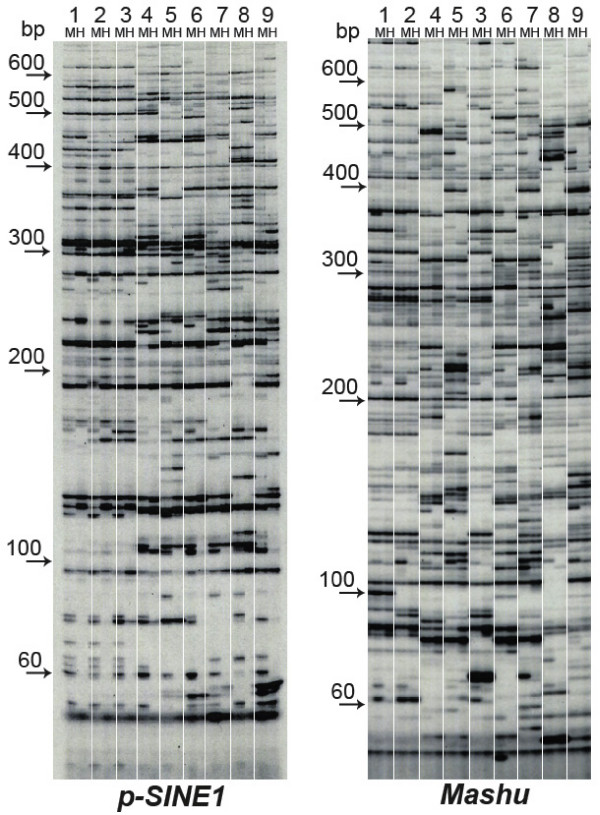
**Transposon displays for *p-SINE1 *and *Mashu *in the nine cultivated and wild rice strains**. The rice strains consist of A58 (no. 1), T65 (no. 2), Nipponbare (no. 3), IR36 (no. 4), Kasalath (no. 5), #108 (no. 6), W107 (no. 7), W593 (no. 8), and W630 (no. 9). In each strain, the two TD samples digested with *Msp*I (M) and *Hpa*II (H) were loaded and electrophoresed in a 50% polyacrylamide gel. Methylated fragments are denoted by the fact that a fragment of the *Msp*I-digested sample has no corresponding fragment in the *Hpa*II-digested sample. The sizes from 60 bp to 600 bp are indicated by the arrows.

Comparison of the TD patterns for the *Hpa*II and *Msp*I-digested samples allowed us to estimate the proportions of the methylated fragments flanked by the 5' end of the TEs (see Figure 3). Figure 2 shows the average proportions of methylated fragments in the total *MspI *fragments of the nine strains. The small standard deviations found in *noaCRR1*, *p-SINE1*, and *Mashu *validate the efficacy of the TD method as capable of detecting methylated fragments equivalently in the different strains. The highest proportions of methylated sequences (over 50%) were observed in the flanking regions of *noaCRR1*, *RIRE5*, *RIRE7*, and *Akan*. These results were appropriate because the *noaCRR1 *family and *RIRE7 *elements are concentrated in the centromeric or pericentromeric regions in rice, where the genomic DNA is heavily methylated [15,16,28]. The other LTR element, *RIRE5*, which is not specifically localized in centromeric or pericentromeric regions, showed a similar tendency to the above LTR elements. These results for the LTR elements agreed with those obtained by high-throughput methods that revealed TEs account for a high proportion of the heavily methylated genomic fraction [5,6,9]. *Akan *is the only class II element that resides in heavily methylated fractions that might be related to heterochromatic regions, but it is localized neither in centromeric nor pericentromeric regions according to BLAST searches (data not shown).

In contrast, the flanking sequences of *Mashu *(Figure 3) and *Basho *were hypomethylated (under 20%) in all nine genomes and showed very small standard deviations. Moderately methylated sequences (around 30%) were found in the flanking sequences of *Kiddo *and *Toya*. Those of the copies of *p-SINE1*, *KiseruI*, *mPing*, and *TabitoII *had similar methylation levels to the average methylation state of the whole genome. Thus, individual TEs reside in chromosomal regions with varying levels of methylation, although stable methylation states of the TE flanking sequences were observed within the same TE family. In the class II elements, the proportions of the methylated fragments differed widely, ranging from that of *Akan *to that of *Mashu*. Like *noaCRR1 *and *RIRE7*, the other individual TE families were present in particular preferential methylation environments of the *Oryza *genomes.

### Relationships with copy number

*Mashu *has more than 500 copies in the rice genome (Table 1 and Figure 3). Despite the large copy number, *Mashu *is not an old element, because the copy organization is relatively conserved and the insertion sites are highly polymorphic between Japonica and Indica strains [29]. The hypomethylation environment around the copies of *Mashu *suggests that the copy number of this TE does not influence the methylation state of the adjacent sequences. The sequence of *Mashu *itself might be associated with the low methylation state, as are the surrounding sequences. It is not necessarily the case that a TE with an abundant copy number is correlated with a high methylation state. TE activities might not always be controlled by DNA methylation, and instead may be controlled in alternative manner(s) such as by gap repair [30,31] or unknown host function(s) to prevent harmful behaviors by TEs. *Akan*, *mPing*, and *Toya *have different methylation states in the surrounding sequences among the nine strains, as shown by the large standard deviations (Figure 2). These are low copy number elements (Figure 1), for which it might be difficult to establish stable methylation states around the elements. Besides the low copy number, the instability of the methylation degree around *mPing *might be due to some activity of the element, which enables it to be inserted into various methylation environments in the genome. Unfortunately, the other active elements in the rice genome were unsuitable for TD analysis due to their very low copy numbers (*Tos17 *[32] and *Karma *[33]) and the presence of a *Msp*I/*Hpa*II site at the terminal of the element (*nDart *[34,35]). The other class II elements, which should be immobilized or very low active elements, were embedded in relatively stable methylation states of the genomic sequences.

### Relationships with conservation degree

The degree of conservation is a rough indication of copy divergence in a TE family (Table 1). Old TE family members tend to have a low degree of conservation and divergent copy organization, while new family members might have better-conserved copy constituents. The conserved elements may be active, like *mPing*, which was the only active class II element in this study [20-22]. Hence, the degree of conservation is certainly related to the activity of the TE element. Here, we tested whether there is a correlation between TE conservation and methylation around the TE. As shown in Table 1 and Figure 2, we did not find a particular correlation between them. This implies an independence of the TE copy organization from the methylation environment in the genome. However, we do not know whether the methylation environment in the genome affect the activity of the TE elements.

### Spread of methylation to the adjacent sequences

**Figure 4:**
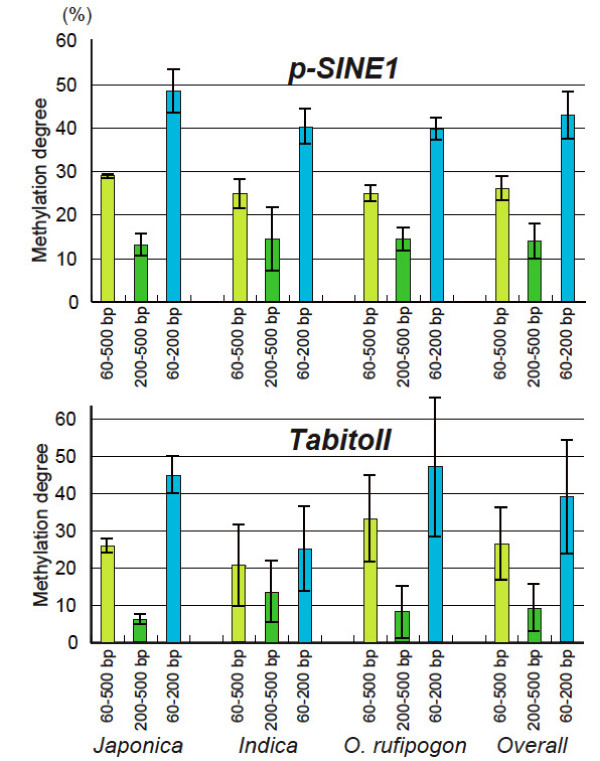
**Differential methylation degrees dependent on the distance from the copies of *p-SINE1 *and *TabitoII***. Based on the band sizes in the TD profiles of the *Msp*I-digested samples, the fragments were divided into two groups: one ranging from 60 to 200 bp and the other ranging from 200 to 500 bp. Methylation degrees were estimated for the fragments in these two ranges and the fragments in the full range from 60 to 500 bp. Within a 500-bp range from the elements, the proximal regions of both *p-SINEI *and *TabitoII *are markedly methylated compared with the distal regions. This tendency was also found in the individual rice strains, as illustrated for Japonica (including A58, T65, and Nipponbare), Indica (IR36, Kasalath, and #108), and *O. rufipogon *(W107, W593, and W630) along with the overall degrees.

When the TD fragments of the *Msp*I-digested samples were sorted into those with distances from the element below 200 bp and above 200 bp, the degrees of methylation were found to be different in the proximal and distal regions of *p-SINE1 *and *TabitoII *(Figure 3 and 4). These proximal regions showed an even heavier methylation state than the distal regions. It seems likely that the methylation state of the TE has spread to the neighboring sequences after insertion. If so, the methylation state of the TE itself should be linked with that of the adjacent sequences. The higher degrees of methylation of sequences within 200-bp of *p-SINE1 *and *TabitoII *might reflect the hypermethylation state of the elements (Figure 4). In fact, *p-SINE1 *copies in the rice genome were strongly suppressed by methylation, whereas *p-SINE1 *could be transcribed when the methylated state was cancelled by anti-sense genes homologous to *Arabidopsis DDM1 *or by 5-azacytidine (Ohtsubo *et al*. unpublished data). In a transgenic rice line retaining a β-glucuronidase reporter gene, the methylation initially occurred in its promoter region derived from the rice pararetrovirus (rice tungro bacilliform virus: RTBV) and gradually expanded to the β-glucuronidase reporter coding sequence in subsequent generations of homozygous offspring [36]. In fact, the rice genome contains about 30 methylated sequences homologous to RTBV [37], which might induce methylation from the promoter to the reporter coding sequence. In fission yeast, the spread of heterochromatin requires read-through transcription to generate RNA interference (RNAi) via small interfering RNAs (siRNAs) [38,39]. Similarly, the spread of DNA methylation might require read-through transcription for the primary substrates. As in the case of the LTR elements where the flanking regions are methylated, the methylated sequence could force methylation of the nearby low-methylated sequences. On the other hand, most of the class II elements examined here were present in low-methylated regions. These elements produce few transcripts corresponding to their genomic sequences, because no well-matched transcripts or ESTs were deposited in the databases (data not shown). Therefore, the methylation states of these elements should be as low as their surroundings if methylation is dependent on siRNAs. Alternatively, it is also possible that the methylation states of the surroundings have influenced those of the elements.

### TEs characterized by degree of methylation of the surroundings

Target site preferences consisting of only a few base pairs have been identified in certain types of MITEs [40-42]. However, it is still difficult to predict the insertion sequences of TEs in the genome even if the known flanking sequences are available, since these sequences show no consensus. However, as shown by Figure 2, TEs are nicely characterized by the degree of methylation of the TE flanking sequences. Although we do not know yet whether a TE can select an insertion site based on the methylation state, our results clearly show that each TE family is characterized by the methylation state of its surroundings. This is the first report demonstrating that TEs in the genome are associated with a particular methylation environment, which is a feature of a given TE.

## Conclusion

TD analysis facilitated a comparison of the methylation degrees among different TEs. In the rice genome database, the 12 TEs analyzed here showed different features of chromosomal distribution, copy number, and conservation degree within a cognate family. The TD analyses revealed that the individual TE families reside in different genomic segments with varying degrees of methylation, but the methylation environments are mostly similar within a given family. The sequences in the vicinity of retrotransposons tend to have a heavier methylation state than those of transposons. A markedly lower methylation state was seen for the flanking sequences of *Mashu *and *Basho*. We failed to find a relationship between the copy number of a TE and the methylation state of the adjacent sequences. Our results also detected no correlation between TE conservation and the methylation state around the TE. In the cases of *p-SINE1 *and *TabitoII*, the degree of methylation decreased with increasing distance from the elements. If most TEs are methylated, as demonstrated by high-throughput analyses [5-7,10], the methylation state of a TE itself might not be simply associated with that of its surroundings. Our results demonstrate that rice TEs are localized in genomic environments with different degrees of methylation characteristic of the particular TE.

## Methods

### BLAST and BLAT analyses

In order to investigate the copy numbers and degrees of conservation of the 12 TEs, we used the BLAST website [27] specified for the rice genome at NCBI. This BLAST analysis considered gaps between the query and subject. All the queries used were representative sequences that were given accession numbers or were selected from reference papers. The other online genome analysis called the Rice Annotation Project DataBase (RAP-DB) developed by the National Institute of Genetics, Japan, can find perfect sequence matches of 33 bases, and sometimes down to 20 bases. The chromosomal locations of the elements can also be searched for by using RAP-BLAT [43]. This system does not consider gaps between the query and subject.

### Plant materials and genomic DNA extraction

The nine rice strains used in these experiments were as follows: A58, T65, and Nipponbare (*Oryza sativa*, Japonica), IR36, Kasalath, and 108 (*O. sativa*, Indica), W107 and W630 (*O. rufipogon*, annual), and W593 (*O. rufipogon*, perennial). These strains are closely related species that can be cross-pollinated with each other. They were maintained as inbred strains by several selfings. In order to obtain genomic DNA with uniform genomic environmental conditions for methylation, all the seeds were sown at the same time and the plants were grown for 3 weeks at about 25°C. Total genomic DNA was isolated from the above-ground parts of the plants (excluding roots), as described by Nagano *et al*. [18].

### Amplified fragment polymorphism (AFLP) and transposon display (TD) to analyze methylation sites

In order to investigate the average frequencies of the methylation-sensitive sites in the rice and related genomes, we used an AFLP method that was basically similar to that reported by Takata *et al*. [14]. The isoschizomers *Hpa*II and *Msp*I were used instead of *Mse*I and *Taq*I as 'frequent cutter' enzymes. Both *Hpa*II and *Msp*I recognize the same tetranucleotide sequence (5'-CCGG-3'), but exhibit differential sensitivity to DNA methylation. *Hpa*II is sensitive to the methylation of any cytosine of 5'-CCGG-3', whereas *Msp*I cuts 5'-C^5m^CGG-3', but not 5'-^5m^CCGG-3'. Thus, 5'-^5m^CCGG-3' or 5'-^5m^C^5m^CGG-3' at the proximal site of TEs may not give band in this TD method. *Eco*RI (5'-GAATTC-3') was employed as the 'less-frequent cutter' enzyme. The oligonucleotides used for adaptors and primers in this study are listed in Additional file 2. All the adaptors were prepared by annealing the two adaptor primers at room temperature after boiling. DNA digestion, adaptor ligation, and PCR reactions were performed according to the method of Takagi *et al*. [29]. Two sets of digestion reactions (*Hpa*II/*Eco*RI and *Msp*I/*Eco*RI) were carried out simultaneously. Approximately 0.25 μg of rice genomic DNA was digested with 5 units of each enzyme in 25 μl of a suitable digestion buffer. Each of the *Hpa*II/*Msp*I and *Eco*RI adaptors (50 μM) was ligated to the digested genomic DNA (200 ng) by the addition of 10 μl of a mix containing 1 × ligation buffer, 0.5 μM ATP, and 1 Weiss unit of T4 DNA ligase and incubation at 16°C overnight. The first PCR reaction was performed in 20 μl of a reaction mixture containing 1 × PCR buffer, 0.2 mM dNTPs (Takara Shuzo Co Ltd., Osaka, Japan), 15 ng of the adaptor-ligated DNA, 10 pmol of each first primer, and 1 unit of EX Taq polymerase (Takara Shuzo Co Ltd.). The first PCR reaction was carried out as follows: 72°C/2 min; 94°C/3 min; 24 cycles of 94°C/30 sec, 54°C/30 sec and 72°C/1 min, and a final cycle of 72°C/5 min. The PCR product was then diluted 20-fold with sterilized water. Nested primers were used for the second PCR: the 5' end of the second primer was labeled with FITC (lambda em = 495 nm), and the 3' end of the second adaptor primers was overhung by three selected nucleotides (ATC, CTC, GAT, GTT or TTA). These five different types of 3' ends of the second adaptor primers were used for each trial (see Additional file 2). The second PCR reaction was performed with the same reaction mixture as the first PCR plus 2 μl of 50% DMSO. For temperature cycling, we modified the conditions used in the "touchdown method": 94°C/5 min, then 10 cycles beginning with 94°C/30 sec, 68°C/30 sec, 72°C/1 min, followed by a reduction of the annealing temperature of the first cycle (68°C) with a 1°C increment per cycle, and then 27 cycles at an annealing temperature of 58°C, and a final incubation at 72°C/5 min. The reaction products were electrophoresed on a 3.5% denaturing polyacrylamide gel containing 50 ml of a mixture of 3.5 ml of Long Ranger (Takara Shuzo Co Ltd.) (equivalent to 50% polyacrylamide:bisacrylamide solution = 19:1) and 7.0 M urea. Internal Lane Standard 600 (Promega, Madison, Wisconsin, USA) was used as the size marker from 60 bp to 600 bp, which was labeled with carboxy-X-rhodamine (lambda em = 576 nm). After the gel was pre-run using 1 × TBE as running buffer, the samples were electrophoresed at a constant 1700 V for 80 min. The fragments and size markers were visualized by scanning the fluorescent signals in the gel plate using a Typhoon 8600 scanner (Molecular Dynamics Inc. Sunnyvale, California).

TD analyses were applied for detection of methylation in the flanking sequences of the 12 TEs. The TD basically followed the AFLP method described above with the following modifications. In order to design the primers in the 5' side of the individual TE sequences, we made consensuses for 60 bp of the 5' end sequences of the individual TEs after surveying sequences homologous to the representative TEs using RAP-BLAT [43] (see Additional file 1). Based on the consensus for each element, nested primers for the TEs were designed, and the 5' end of the second TE primer was labeled with FITC (see Additional file 2). No overhung nucleotide was added at the 3' end of the second adaptor primers in the TD method. In the cases of LTR elements, the PCR reactions elongate to both the outside and inside of the elements, because the primer sequences reside in the tandem repeats of the LTR elements. A very limited number of the TD bands were directed to the internal sequence of LTR elements due to its higher conservation among the copies compared with the flanking sequences. Thus, we did not take these bands away from the total band numbers when the proportions of methylated bands were calculated.

## List of abbreviations

AFLP – amplified fragment length polymorphism

MITE – miniature inverted repeat transposable element

LINE – long interspersed nucleotide element

LTR element – long terminal-repeat retrotransposon

SINE – short interspersed nucleotide element

TD – transposon display

TE – transposable element

## Authors' contributions

MK, AK, and AT conceived the experiments and carried out the molecular analyses and databases searches. YK conceived of the study and drafted the manuscript. HO participated in the sequence alignment, database analysis, and the design of the study. YK and YS participated in the basic design of this study and coordination. All authors read and approved the final manuscript.

## Supplementary Material

Additional file 1 The 60-bp consensus sequences of the 5' end of 12 TEs. The data provided represent the 60-bp consensus sequences determined from the rice database for the 5' end of 12 TEs. Click here for file

Additional file 2 Primers used in this study. All the primer sequences used in this study are provided. Click here for file
