# Supplementary material for: Rice transposable elements are characterized by various methylation environments in the genome
Source: BMC Genomics. 2007 Dec 20;8:469. doi: 10.1186/1471-2164-8-469 (PMC2222647; doi:10.1186/1471-2164-8-469)
Supplement: Additional file 1 — The 60-bp consensus sequences of the 5' end of 12 TEs. The data provided represent the 60-bp consensus sequences determined from the rice database for the 5' end of 12 TEs. [file 1471-2164-8-469-S1.pdf]

Additional file 1 The 60-bp consensus sequences of the 5' end of 12 TEs

TE	Consensus sequences	Sequence no. (1)
noaCRR	TGATGTGACCATGGCTACTACGGATACAACCATTGCTCACATCATGAATCAACAAGAAGA	63
RIRE5	TGTTAGATTAATGGGCTAGGCCCAATTAAATCCTAATAAATTCCATTGGCCCACATTAAG	69
RIRE7	TGATGAGGACATCCCTTCCAACGATACAACCACGCCTATTGCACAGCAAGGACCAATGAC	38
p-SINE	GAGAAATGCCCAGGGGTCTTCCGGCTAGCTCCACAAGGTGGTGGGCTAGACGACCTGGGT	30
Akan	CAAGGTTGTCAGTATCCCGATTCGTATCCTAGTATCTTACGATACTACGATCCTACCAAG	19
Kiddo	CTCCCTCCGTACTCGTAAAGGAAGTCGTTTAGGACAGCGACACGGTCTCCAAAACACAAC	24
KiseruI	GGGGCTGTTTGGTTCCCAGCCACACTTTACCATTACTTGCCAACAAAAGTTGCCACACCT	63
mPing	GGCCAGTCACAATGGGGGTTTCACTGGTGTGTCATGCACATTTAATAGGGGTAAGACTGA	35
Mashu	GAGCACCCGCAATGGTAAAGTAAGGTGCTATCTATAAAACATGTACATCTCAGCAATAGA	100
TabitoII	GGCCGTGTTTAGTTCCAAACTTTTTCTTCCAAACTTCAACTTTTCCATCACATCAAAACT	100
Toya	CAGTGGCGGATTTAGGCCATTTCTGTGGGGTCGGCTGACCCCACAGCTTTTTGAAAAACA	11
Basho	CTAGCATGGTGGCCCGCGCAGATTGCGCGGCTAGCATCATTATATTTTCTCTCATATAAT	40

1: The numbers of sequences obtained from the rice databases to prepare the consensus sequences.
