# Supplementary material for: Rice transposable elements are characterized by various methylation environments in the genome
Source: BMC Genomics. 2007 Dec 20;8:469. doi: 10.1186/1471-2164-8-469 (PMC2222647; doi:10.1186/1471-2164-8-469)
Supplement: Additional file 2 — Primers used in this study. All the primer sequences used in this study are provided. [file 1471-2164-8-469-S2.pdf]

Additional file 2 Primers used in this study

Name of primer	Sequence(5'→3')
HpaII/MspI adaptor 1st	GTAATACGACTCACTATAGGGC
HpaII/MspI adaptor 2nd	GTGCTTGATGCTTGAAAACGG
noaCRR 1st	CAATGGTTGTATCCGTAGTAGC
noaCRR 2nd	AATGGTTGTATCCGTAGTAGCC
RIRE5 1st	AATTGGGCCTAGCCCATTAATC
RIRE5 2nd	GGGCCTAGCCCATTAATCTAAC
RIRE7 1st	AATAGGCGTGGTTGTATCGTTGG
RIRE7 2nd	TGGTTGTATCGTTGGAAGGGATGTC
p-SINE 1st	TGTGGAGCTAGCCGGAAGAC
p-SINE 2nd	AGCTAGCCGGAAGACCCCTG
Akan 1st	ATCGTAAGATACTAGGATAC
Akan 2nd	ACTAGGATACGAATCGGGATAC
Kiddo 1st	CAAGTAATGGTAAAGTGTGGCTGGG
Kiddo 2nd	AATGGTAAAGTGTGGCTGGGAAC
Kiseru I 1st	TCGCTGTCCTAAACGACTTC
Kiseru I 2nd	TGTCCTAAACGACTTCCTTTACG
mPing 1st	ACACCAGTGAAACCCCCATTG
mPing 2nd	AGTGAAACCCCCATTGTGACTG
Mashu 1st	ATAGATAGCACCTTACTTTACCATTG
Mashu 2nd	CCTTACTTTACCATTGTGGGTGCTC
TabitoII 1st	GATGGAAAAGTTGRAAGTTTGAAGAAAAAG
TabitoII 2nd	TTGAAGAAAAAGTTWGGAACTAAACWCGGC
Toya 1st	CACAGAAATGGCCTAAATCC
Toya 2nd	CAGAAATGGCCTAAATCCGC
Basho 1st	GAGAGAAAATATAATGATGCTAGCCG
Basho 2nd	AATCTGCGCGGGCCACCAT
